# Kinetic analysis of the metatarsophalangeal joint in normal subjects and hallux valgus patients during walking using a four-segment foot model

**DOI:** 10.1186/1757-1146-7-S1-A125

**Published:** 2014-04-08

**Authors:** Bora Jeong, Seunghyeon Kim, Jongsang Son, Youngho Kim

**Affiliations:** 1 Yonsei University, Wonju, Gangwon, 220-710, Korea

## 

The foot plays an important role in human walking [1]. The foot has many essential functions such as shock absorption, weight bearing stability and push-off. The metatarsophalangeal (MP) joint, positioned between the metatarsal bones of the foot and the proximal phalanges of the toes, provides a broad area of support across the forefoot. The major role of the MP joint is the energy absorption during the terminal stance of the gait cycle [2].

Hallux valgus (HV), the most common great toe disorder, is a deformity of the first MP joint [3]. In HV patients, the mechanical role of the MP joint might change to compensate for the worsening of the loading condition, decreased weight-bearing function of the medial toe, and weight transfer to the lateral metatarsals [4-7]. Some researchers have investigated kinematics of the MP joint [8,9]. However, there was rare investigation of the MP joint kinetics. In this study, kinetics of the MP joint was determined during the entire stance period of the gait cycle using a four-segment foot model.

The three-dimensional motion analysis was used with foot pressure measurement. Twelve normal subjects and ten HV patients were selected for this study.

Results showed that a significant difference in stance time was found between the normal (60.86 ± 1.21 %) and HV groups (63.75 ± 0.91 %) (*p* < 0.05). The ankle joint moment for the normal group and the HV group was not significantly different. However, the peak MP1 moment in the HV group was significantly smaller than in the normal group (*p* < 0.05). Considerable energy absorption was observed from the terminal stance to pre-swing in both groups. However, total energy absorption in all MP joints decreased 25% in the HV group (4.59 ± 0.85 J/kg) compared with the normal group (6.09 ± 1.00 J/kg). The energy absorption in the MP1 joint and the MP2 joint were significantly smaller in the HV group than in the normal group (*p* < 0.05). However, no significant difference in energy absorption for the MP3−5 joint was observed between the normal group and the HV group (*p* > 0.05).

This study had some limitation such as assumption that the MP3−5 joints act as a single joint and small number of the HV patients. In spite of those limitations, our study would be helpful in understanding the mechanical role of the MP joint in patients with foot disease.

**Figure 1:**
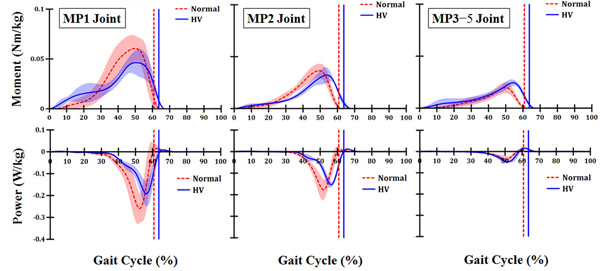
The MP1, MP2 and MP3−5 joint moments and powers; Normal vs. HV
